# Unusual Case of External Juxta Coronal Odontoma

**DOI:** 10.30476/DENTJODS.2020.85652.1142

**Published:** 2021-12

**Authors:** Fatemeh Dehghani Nazhvani, Ali Azadikhah, Tayebeh Dorudizadeh, Pardis Haddadi, Ali Dehghani Nazhvani, Nafiseh Shamloo

**Affiliations:** 1 Bone and Joint Diseases Research Center, Shiraz University of Medical Sciences, Shiraz, Iran; 2 Postgraduate Student, Dept. of Periodontology, School of Dentistry, Shiraz University of Medical Sciences, Shiraz, Iran; 3 Postgraduate Student, Dept. of Pediatric Dentistry, School of Dentistry, Shiraz University of Medical Sciences, Shiraz, Iran; 4 Dept. of Periodontology, School of Dentistry, Lorestan University of Medical Sciences, Khorramabad, Iran; 5 Dept. of Oral & Maxillofacial Pathology, Biomaterials Research Center, School of Dentistry, Shiraz University of Medical Sciences, Shiraz, Iran; 6 Dept. of Oral & Maxillofacial Pathology, School of Dentistry, Shahid Beheshti University of Medical Sciences, Tehran, Iran

**Keywords:** Odontoma, Case report, Juxtacoronal position

## Abstract

Odontomas are benign tumors of jaws with mixed tissue, which are the result of proliferation of odontogenic epithelium and mesenchymal cells. They occur almost centrally and seldom peripherally. There is no report of such a lesion externally while attaching a tooth crown. In this case, we present a lesion on the buccal surface of the right maxillary central incisor crown, which is misconstrued with a dental overgrowth. Such cases may confuse diagnosis during clinical examination. Radiographically, such odontomas may be mistaken for various other lesions. Ultimate diagnosis should be relied upon microscopic evaluation and histopathological results.

## Introduction

Odontomas are benign lesions with mixed tissue, which are the result of proliferation of odontogenic epithelium and mesenchymal cells. Unlike real neoplasms, odontomas are most likely hamartomas [ 1 ]. Based on their similarity to normal tooth structure, microscopic and radiographic views, odontomas are subdivided into compound (small tooth like structures) and complex (a mass of enamel, dentin, and variable amount of cementum) [ 2 ].

The accurate etiology of odontomas is still unclear, but infection, local trauma, genetic factors, or family history of some syndromes such as Gardner syndrome and Hermann syndrome can cause odontoma [ 3 ]. The most common teeth affected by odontoma are canines, incisors and third molars, respectively [ 4 ]. They occur almost centrally and seldom peripherally [ 3 - 4 ]. 

In the literature, there is no report of such a lesion externally while attaching a tooth crown. This location is attributed to dental overgrowths such as enamel pearl, bifurcation ridge, and talon cusp, which have an organized structure that includes, enamel, dentin, and sometimes pulp in order that we often see in a normal tooth structure [ 5 - 6 ].

In this case, we present a lesion on the buccal surface of the right maxillary central incisor crown, which is not completely similar to the above lesions.

## Case Presentation

**Figure 1:**
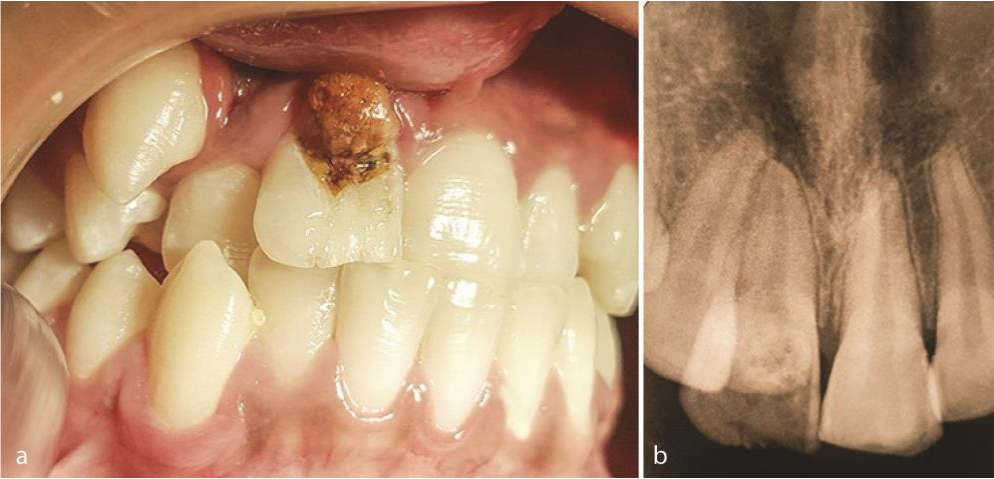
**a:** Abnormal shape of the right maxillary central incisor crown, **b:** X-ray view: combination of radiolucent and radiopaque lesion with no periapical change

A 14-year-old male presented with expressed concern about the abnormal shape of the right maxillary central incisor crown (Figure 1a). His main complaint was abnormal tooth shape and impaired aesthetics. There was no particular medical history. No similar dental abnormalities were seen in other members of the family. An intra-oral examination revealed good dental health. The occlusion was a class I molar relationship but there was a severe lack of space.

The right maxillary central incisor had a lesion that was first looked like calculus. The consult with periodontists revealed that it was not calculus. The lesion was extended to the cervical part of crown and root.

The size of lesion was about 8 mm. The patient had no pain and no clinical symptom. It was not associated with caries and vitality tests revealed normal pulp response. No association with other dental anomalies was established. A periapical radiograph was provided (Figure 1b). On radiological examination, the lesion presented a combination of radiolucent and radiopaque appearance and no periapical changes were noticed. 

**Figure 2:**
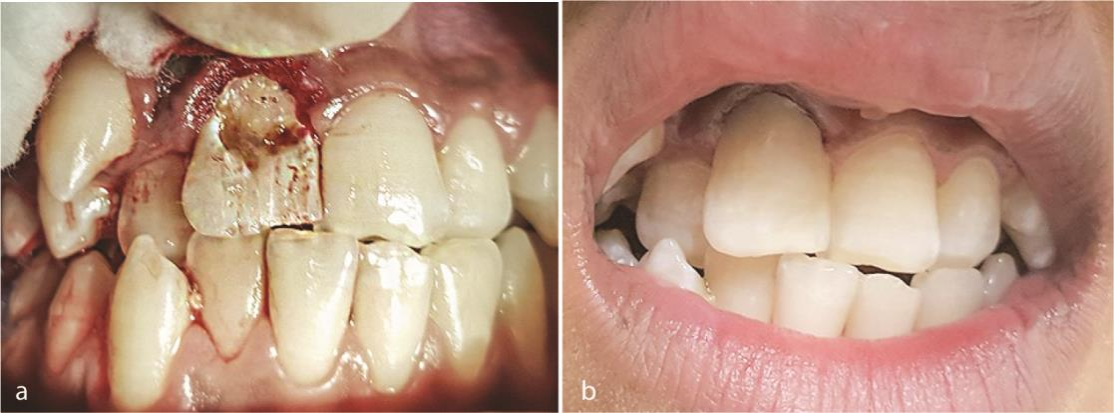
**a:** Therapeutic procedure included complete removal of the lesion, **b:** Aesthetic correction with composite material

**Figure 3:**
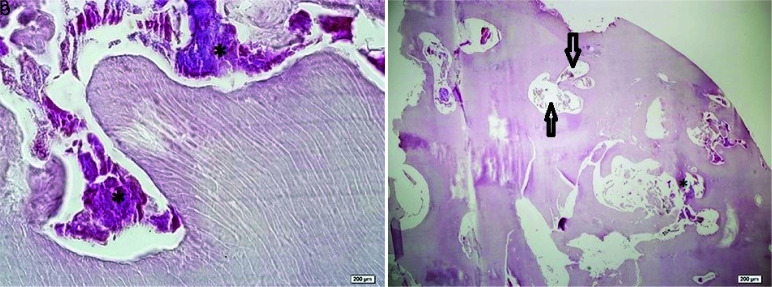
Histopathological sections showed tubular dentin and enamel matrix (Asterisks) (**a:** H&E stain, 100X) in an unorganized manner with flashes introducing pulpal connective tissue and an asterisk presenting cementum (**b:** H&E stain, 400X) as we see in a complex odontoma

Initially, it was attempted to remove the lesion with an ultrasonic scaler; but it was not possible. So finally, therapeutic procedure included complete removal of the lesion (Figure 2a) with fine diamond burr in a high speed handpiece and aesthetic correction was performed with composite material immediately after removal of the lesion and managing the bleeding (Figure 2b). The resected material was put in formalin and sent for histopathological evaluation. The microscopic data showed the lesion was composed of tubular dentin and enamel matrix in an unorganized manner as we see in a complex odontoma (Figure 3a and b). Patient was followed up for six months and no recurrence was reported. Informed consent was read and signed by the patient for publishing his images and data anonymously. This report was confirmed by Research Ethic Committee of Shiraz University of Medical Sciences, Shiraz, Iran (code: IR.SUMS.DENTAL. REC.1399.109).

## Discussion

Odontoma is a benign odontogenic lesion. They are probably a hamartomatous malformation of functional ameloblasts and odontoblasts rather than true neoplasms [ 2 ]. They usually include dentin, enamel, different amounts of cementum, and pulp tissues [ 7 ].

The exact etiology of odontomas is unclear, different factors such as local trauma, infection, growth pressure, heredity and developmental influences may be the causes [ 1 , 8 - 9 ].

Odontomas may be revealed at any age, but their highest prevalence is in the first and second decades of life with a slight male tendency [ 2 ]. The case presented is also in second decade of his life. These tumors occur almost centrally and seldom peripherally specially in the posterior of mandible [ 3 ]. Compound odontomas are most commonly seen in the anterior of the maxilla, whereas complex odontomas are most commonly seen in the posterior of mandible. Odontomas are not frequently associated with the primary teeth [ 4 , 10 - 11 ]. They are prevalent in children and adolescents [ 12 ]. Most tumors are found on routine radiological examinations. The canines, then upper central incisors and third molars, are the most common areas for odontomas [ 4 ]. Interestingly, both types of odontomas occur more frequently on the right side of the jaws than on the left, similar presentation was seen in our case [ 7 ].

Radiographically, they generally appear as small, solitary, or multiple mixed radiolucent-radiopaque lesions. Complex odontoma appears as an irregular mass of calcified material surrounded by a thin radiolucent area with smooth periphery and the compound type shows calcified structures resembling teeth in the center of a well-defined radiolucent lesion [ 4 ]. The irregular radiopaque view of this case evoked the idea that the mass had the density similar to the teeth. 

Compound odontomas are tooth like structures. Their histopathologic feature looks like pulp tissue in the center, surrounded by a dentin shell and then enamel matrix. Complex odontomas are disorganized combination of dentin, enamel, cementum, and areas of pulp tissue without orientation; the current case had similar structure [ 4 ]. Although there are cases of erupted odontoma in the literature as proposed by Rumel *et al*. [ 13 ] for the first time in 1980 and few erupted odontomas reported by Serra in 2009 [ 14 ]; no reported case was found similar to our case as an external juxtacoronal mass. 

Vengal *et al*. [ 15 ] indicated that most of erupted odontomas occur in people younger than 40 years. Moreover, Hanemann *et al*. [ 16 ] reported that peripheral odontoma arising in the extraosseous soft tissues is rare and if not removed early, may enlarge over time and eventually exfoliate. The case presented here was not a peripheral one because no mucosa had covered it.

Unlike dental overgrowths, our case did not have a regular structure of enamel and dentin. So based on histopathological results, it evokes an odontoma. Consequently, the case is neither a typical odontoma based on the location, nor a typical overgrowth, so we called it juxtacoronal odontoma. 

## Conclusion

An unusual juxtacoronal odontoma on the right maxillary central incisor region is reported. Such cases may confuse the diagnosis during clinical and radiographical examinations and they might be mistaken for various other lesions. Therefore, the clinicians might rely on microscopic evaluation.

## Conflict of Interest

All authors declare no conflict of interests.
